# Supplementary material for: The broad structure of psychopathology in the All of Us Research Program
Source: Psychol Med. 2025 Nov 14;55:e344. doi: 10.1017/S0033291725102407 (PMC13058667; doi:10.1017/S0033291725102407)
Supplement: Ehteshami and Waldman supplementary material 1 — Ehteshami and Waldman supplementary material [file S0033291725102407sup001.pdf]

Supplemental Online Content

**eMethods**	**p.2**
**eResults**	**p.3**
**eReferences**	**p.6**
**eTable 1.** Names and concept codes for initial set of selected diagnoses	**p.9**
**eTable 2.** Frequencies and diagnostic codes for psychiatric conditions used in analyses	**p.12**
**eTable 3.** All of Us sample characteristics across race-ethnicity	**p.13**
**eTable 4.** Fit and alternative indices for confirmatory and exploratory models	**p.15**
**eTable 5.** Alternative indices for fear and distress diagnoses across confirmatory models	**p.17**
**eTable 6.** Fit and alternative indices for bifactor and modified bifactor versions of best fitting models	**p.18**
**eTable 7.** Comparisons among factors in Model 9.5 in their alternative indices	**p.19**
**eTable 8.** Split-half replication of all models	**p.20**
**eTable 9.** Split-half replication of exploratory bifactor models	**p.22**
**eTable 10.** Split-half replication of exploratory modified bifactor models	**p.23**
**eTable 11.** External correlates of the higher-order psychopathology dimensions	**p.24**
**eFigure 1.** Prevalences of the 39 primary lifetime diagnoses	**p.25**
**eFigure 2a.** Correlations of D, F, N, and T factors with Distinct Externalizing, Substance Use, and Combined Externalizing + Substance Use factors	**p.26**
**eFigure 2b.** Correlations of E, N, S, and T factors with Fear, Distress, and a broad Internalizing factor	**p.26**
**eFigure 3.** Indices of robustness of the results of the best-fitting model using random models	**p.27**

eMethods

Sociodemographic correlates

- Sex assigned at birth: Female, Male
- Annual household income: under $10k, $10k-$25k, $25k-$35k, $35k-$50k, $50k-$75k, $75k-$100k, $100k-$150k, $150k-$200k, more than $200k
- Educational attainment: Less than a high school degree or equivalent; Highest Grade: Twelve or GED; Highest Grade: College One to Three; College graduate or advanced degree

Targeted analyses for ambiguously classified disorders

In addition to these overarching structural hypotheses, we conducted several targeted CFAs and exploratory analyses to address the ambiguous placement of specific disorders within the existing psychopathology literature. These analyses were designed to clarify how certain disorders align with, or deviate from, broader latent dimensions. First, we tested a priori hypothesized correlated residuals among ADHD subtypes to account for any covariation not fully explained by their shared factor loadings. Second, we examined whether ADHD and its subtypes are best conceptualized as part of the Externalizing spectrum, the Neurodevelopmental domain, or both. Third, we investigated whether Eating Disorders reflect underlying Internalizing pathology (particularly Distress) and/or Thought Problems. Fourth, we assessed whether Obsessive-Compulsive Disorder (OCD) and Obsessive-Compulsive Personality Disorder (OCPD) load on Neurodevelopmental Disorders, Internalizing, Fear, Distress, and/or Thought Problems, reflecting ongoing debate about their nosological placement. Fifth, we evaluated whether Tics and Tourette's Syndrome align more closely with Neurodevelopmental Disorders or Distress. Sixth, we tested whether Antisocial Personality Disorder (ASPD) is best represented by Externalizing, Substance Use, or both (Verona, Javdani, & Sprague, 2011; Voorhees et al., 2014). Seventh, we explored whether Borderline Personality Disorder (BPD) reflects Distress, Thought Problems, and/or Externalizing, given its complex and multifaceted clinical presentation.

To further evaluate potential model misspecifications, we introduced theoretically and empirically justified correlated residuals between closely related disorders, including ADHD subtypes, OCD with OCPD, Schizophrenia with Schizoaffective Disorder, and Major Depressive Disorder (MDD) with Dysthymia. Finally, in an empirically driven effort to refine model fit, we used Modification Indices (MIs)—which quantify expected model improvement based on the change in c^2—to guide the addition of correlated residuals and cross-loadings. Importantly, cross-loadings were added only when there was clear theoretical and/or empirical support in the existing literature. Given the known risks of overfitting and capitalization on chance inherent in MI-guided modifications (MacCallum, Roznowski, & Necowitz, 1992), we evaluated all added parameters using alternative fit indices and tested their robustness through split-half replication procedures.

Statistical analyses

Our approach involved conducting factor analyses on a continuum ranging from fully confirmatory to more exploratory, using Confirmatory Factor Analyses of the binary diagnoses. We employed the Weighted Least Squares Means and Variance Adjusted Satterthwaite estimator (WLSMVS)(Flora & Curran, 2004; Muthén, 1984; Shi, DiStefano, McDaniel, & Jiang,

2018) in the R (4.4.0) package *lavaan* (0.6-17)(Loehlin & Beaujean, 2017; Rosseel, 2012) on the *All of Us* Workbench to test and replicate more comprehensive models of psychopathology than was previously possible.

Our tested models represent a substantial subset of the Hierarchical Taxonomy of Psychopathology (HiTOP)(Conway, Forbes, South, & Consortium, 2021; DeYoung et al., 2021; Kotov et al., 2017, 2021; Krueger et al., 2021; Ringwald, Forbes, & Wright, 2023), an overarching framework supported by extensive research. We tested several major components of this model, focusing on the higher-order dimensions of Externalizing (E), Internalizing (I), Distress (D), Fear (F), Substance Use Disorders (S), Thought Problems (T), and Neurodevelopmental Disorders (N). In addition to contrasting *a priori* models, we used modification indices to search for cross-loadings that might enhance model fit.

To assess model fit, we employed multiple indices given their desirable properties: the Comparative Fit Index (CFI)(Bentler, 1990), Tucker-Lewis Index (TLI)(Tucker & Lewis, 1973), Root Mean Square Error of Approximation (RMSEA) with its 95% confidence interval(Browne & Cudeck, 1992), and Standardized Root Mean Squared Residual (SRMR)(Bentler, 1995; Loehlin & Beaujean, 2017). Standard guidelines representing adequate and good fit for these fit indices are CFI and TLI values > .90 and > .95, respectively; RMSEA values of < .08 and < .05, respectively; and SRMR < .08(Li-tze Hu & Bentler, 1998; Li-tze Hu & Bentler, 1999). Researchers have recently documented the limitations of conventional fit indices (e.g., overfitting, differential fit propensity, and bias in tests of certain models) for adjudicating among structural models of psychopathology and have suggested alternative criteria(Bonifay & Cai, 2017; Bonifay, Lane, & Reise, 2017; Forbes et al., 2021; Greene et al., 2019; Morgan, Hodge, Wells, & Watkins, 2015; Murray & Johnson, 2013; Watts, Poore, & Waldman, 2019). Hence, we augmented these fit indices with additional indices for adjudicating among alternative models: the mean, median, standard deviation (SD), and standard errors (SE) of standardized factor loadings, as well as the sensitivity of factor loadings to each factor's constituent diagnoses. Superior models are characterized by higher mean and median factor loadings with lower SEs and SDs, as well as factor loadings that are less sensitive to the removal of any single diagnosis(Waldman et al., 2022). We anticipated that these alternative indices would provide incremental utility beyond conventional fit indices in discriminating between competing models.

eResults

Distinguishing Fear and Distress within Internalizing

Several models showed similar overall fit, particularly the model treating Internalizing as a single dimension compared to the model splitting it into Fear and Distress (Hypothesis 2: Models 5 vs. 9). Both models had identical fit indices but differed in alternative indices for relevant diagnoses (shown in eTable 5 in Supplement 1). For Distress-related diagnoses, median and standard deviations of factor loadings were similar between models (.545 and .121 for Model 5 vs. .547 and .140 for Model 9), though Model 5 had lower standard errors (.015 vs. .007). For Fear-related diagnoses, Model 9 demonstrated higher median factor loadings (.591 vs. .535) with slightly greater standard deviations (.298 vs. .268) and standard errors (.022 vs. .020). These results underscore the benefit of distinguishing between Fear and Distress to better capture diagnostic variability. Additionally, as shown in eFigure 2b in Supplement 1, correlations of Externalizing, Substance Use, Thought Problems, and

Neurodevelopmental Disorders with broad Internalizing and Distress were similar, but correlations with Fear were notably lower. Despite a high correlation between Fear and Distress (r = .898, SE = .011), these results indicate that Fear and Distress are distinct dimensions. Consequently, the six-factor model distinguishing these dimensions was identified as the preferred framework for analyzing ambiguously classified disorders.

Correlated factors models vs. bifactor models

A mixed picture emerged when comparing the correlated factors models to their bifactor model equivalents that also contained a general factor (shown in eTable 6). While conventional fit indices favored most bifactor models (given higher CFI and TLI, equivalent RMSEA, and lower SRMR), the alternative indices strongly favored the correlated factors models. Specifically, the correlated factors models demonstrated higher median factor loadings and lower standard deviations and standard errors, and these indices were much less consistent across random halves in the bifactor models. Notably, the 6 correlated factors model outperformed the corresponding bifactor model even according to conventional fit indices. These results further support the 6 correlated factors model (shown in Figure 2) as the best-fitting a priori hypothesized model. Moreover, these findings highlight the utility of alternative indices and factor correlations in augmenting conventional fit indices for adjudicating among alternative structural models of psychopathology.

The Placement of Ambiguously Classified Disorders

We examined the fit and alternative indices of several targeted CFAs to resolve the placement of ambiguously classified disorders (Table 1 in the main text shows results for disorders whose placement could be resolved, whereas eTable 4 in Supplement 1 shows the full set of results).

Differences in fit for the placement of ADHD or its subtypes were small and thus hard to adjudicate (Models 9.1 - 9.6). Having ADHD load on both E and N or having ADHD-Hyperactive-Impulsive subtype (ADHD-HI) load on E and ADHD-Combined subtype (ADHD-C) and ADHD-Inattentive subtype (ADHD-IN) load on N with correlated residuals between ADHD-HI and the other two subtypes, showed similar fit. The latter model (Model 9.5) was preferred, given that the loadings of the ADHD subtypes were substantially higher, commensurate with the other diagnoses' loadings on E and N.

The placement of eating disorders (including Anorexia Nervosa and Bulimia Nervosa; Models 9.5.1 - 9.5.5) could not be definitively resolved, as both model fit and alternative indices similarly favored their placement on either D only, or D and T. There was nearly equivalent support for a model in which anorexia loaded on D and bulimia loaded on D and E.

Despite similarities in model fit and alternative indices, there was some suggestion that OCD and OCPD might reflect F or D, rather than T (Models 9.5.6 - 9.5.9). Results for Tics and Tourette's Syndrome (Models 9.5.15 and 9.5.16) suggested they may be better placed on D instead of (or in addition to) N.

Antisocial Personality Disorder appeared to better reflect S, or S and E, rather than E only, although differences in factor loadings and correlations for these models were minimal (Models 9.5 and 9.5.17). Borderline Personality Disorder appeared to reflect D in addition to T or E, rather than T alone (Models 9.5.10 - 9.5.14).

Based on the high similarity of their diagnoses, eight correlated residuals were considered *a priori* for addition to the preferred 6 correlated factor model (Models 9.5.18 - 9.5.26). When added simultaneously to Model 9.5, three correlated residuals (between schizophrenia and schizoaffective disorder, OCD and OCPD, and MDD and dysthymia) improved model fit while

maintaining equivalent levels of the alternative indices. Finally, Modification Indices increased conventional fit indices only slightly while worsening alternative indices.

Replication and Robustness Across Random Sample Halves and Randomly Assigned Diagnoses

We tested for replication of the model fitting results across random halves of the *All of Us* sample. As shown in eTable 8, findings were highly similar across the random halves and mirrored those from the full sample. Models demonstrated better fit and more favorable alternative indices with increasing model complexity. Specifically, intra-class correlations of the fit and alternative indices across random halves of the sample were very high (.90 to 1.0), except for mean factor loadings (FLs) which had a correlation of .765. Differences across random halves in the indices were minimal, ranging from .009 for FL standard deviations (SDs) to .0026 for the Tucker-Lewis Index (TLI). These indices were much less consistent for the bifactor models across random halves (eTable 9 and eTable 10). Although intra-class correlations of the CFI and TLI were similarly high (.93), these correlations were lower for RMSEA and SRMR (.79 and .85) and for the mean and SD of factor loadings (.56 and .52). Differences in CFI, TLI, RMSEA, and SRMR were minimal (.00009 to .0023), but greater for the mean and SD of factor loadings (.024 and .048).

To address concerns that our preferred six-factor models might have the correct number of factors but incorrect diagnoses loading on each factor, we generated 1,000 random variations of the 6-factor model. In each iteration, we kept the number of diagnoses per factor constant but randomly assigned diagnoses to factors. eFigure 3 displays the fit statistics and alternative indices for these 1,000 randomly generated models, as well as the fit of the preferred 6-factor model. Our preferred model demonstrated substantially better fit according to the fit statistics and had much higher factor loadings (FLs) and much lower standard deviations (SDs) than models with diagnoses randomly assigned to factors. The FLs and SDs of the random models were much closer to those from the bifactor models. Paradoxically, the mean and median standard errors (SEs) of the random models were lower than in our best-fitting model.

eReferences

Bentler, P. M. (1990). Comparative Fit Indexes in Structural Models. *Psychological Bulletin*, *107*(2), 238–246. https://doi.org/10.1037/0033-2909.107.2.238

Bentler, P. M. (1995). *EQS structural equations program manual* (Vol. 6). Multivariate software Encino, CA.

Bonifay, W., & Cai, L. (2017). On the Complexity of Item Response Theory Models. *Multivariate Behavioral Research*, *52*(4), 465–484. https://doi.org/10.1080/00273171.2017.1309262

Bonifay, W., Lane, S. P., & Reise, S. P. (2017). Three Concerns With Applying a Bifactor Model as a Structure of Psychopathology. *Clinical Psychological Science*, *5*(1), 184–186. https://doi.org/10.1177/2167702616657069

Browne, M. W., & Cudeck, R. (1992). Alternative Ways of Assessing Model Fit. *Sociological Methods & Research*, *21*(2), 230–258. https://doi.org/10.1177/0049124192021002005

Conway, C. C., Forbes, M. K., South, S. C., & Consortium, H. (2021). A Hierarchical Taxonomy of Psychopathology (HiTOP) Primer for Mental Health Researchers. *Clinical Psychological Science*, *10*(2), 236–258. https://doi.org/10.1177/21677026211017834

DeYoung, C. G., Kotov, R., Krueger, R. F., Cicero, D. C., Conway, C. C., Eaton, N. R., … Wright, A. G. C. (2021). Answering Questions About the Hierarchical Taxonomy of Psychopathology (HiTOP): Analogies to Whales and Sharks Miss the Boat. *Clinical Psychological Science*, 216770262110493. https://doi.org/10.1177/21677026211049390

Flora, D. B., & Curran, P. J. (2004). An Empirical Evaluation of Alternative Methods of Estimation for Confirmatory Factor Analysis With Ordinal Data. *Psychological Methods*, *9*(4), 466–491. https://doi.org/10.1037/1082-989x.9.4.466

Forbes, M. K., Greene, A. L., Levin-Aspenson, H. F., Watts, A. L., Hallquist, M., Lahey, B. B., … Krueger, R. F. (2021). Three Recommendations Based on a Comparison of the Reliability and Validity of the Predominant Models Used in Research on the Empirical Structure of Psychopathology. *Journal of Abnormal Psychology*, *130*(3), 297–317. https://doi.org/10.1037/abn0000533

Greene, A. L., Eaton, N. R., Li, K., Forbes, M. K., Krueger, R. F., Markon, K. E., … Kotov, R. (2019). Are Fit Indices Used to Test Psychopathology Structure Biased? A Simulation Study. *Journal of Abnormal Psychology*, *128*(7), 740–764. https://doi.org/10.1037/abn0000434

Hu, Li-tze, & Bentler, P. M. (1998). Fit Indices in Covariance Structure Modeling: Sensitivity to Underparameterized Model Misspecification. *Psychological Methods*, *3*(4), 424–453. https://doi.org/10.1037/1082-989x.3.4.424

Hu, Li-tze, & Bentler, P. M. (1999). Cutoff criteria for fit indexes in covariance structure analysis: Conventional criteria versus new alternatives. *Structural Equation Modeling: A Multidisciplinary Journal*, *6*(1), 1–55. https://doi.org/10.1080/10705519909540118

Kotov, R., Krueger, R. F., Watson, D., Achenbach, T. M., Althoff, R. R., Bagby, R. M., … Zimmerman, M. (2017). The Hierarchical Taxonomy of Psychopathology (HiTOP): A Dimensional Alternative to Traditional Nosologies. *Journal of Abnormal Psychology*, *126*(4), 454–477. https://doi.org/10.1037/abn0000258

Kotov, R., Krueger, R. F., Watson, D., Cicero, D. C., Conway, C. C., DeYoung, C. G., … Wright, A. G. C. (2021). The Hierarchical Taxonomy of Psychopathology (HiTOP): A Quantitative Nosology Based on Consensus of Evidence. *Annual Review of Clinical Psychology*, *17*(1), 1–26. https://doi.org/10.1146/annurev-clinpsy-081219-093304

Krueger, R. F., Hobbs, K. A., Conway, C. C., Dick, D. M., Dretsch, M. N., Eaton, N. R., … Workgroup, H. U. (2021). Validity and utility of Hierarchical Taxonomy of Psychopathology (HiTOP): II. Externalizing superspectrum. *World Psychiatry*, *20*(2), 171–193. https://doi.org/10.1002/wps.20844

Loehlin, J. C., & Beaujean, A. A. (2017). *Latent Variable Models, An Introduction to Factor, Path, and Structural Equation Analysis, Fifth Edition*. New York: Taylor & Francis. https://doi.org/10.4324/9781315643199

MacCallum, R. C., Roznowski, M., & Necowitz, L. B. (1992). Model Modifications in Covariance Structure Analysis: The Problem of Capitalization on Chance. *Psychological Bulletin*, *111*(3), 490–504. https://doi.org/10.1037/0033-2909.111.3.490

Morgan, G., Hodge, K., Wells, K., & Watkins, M. (2015). Are Fit Indices Biased in Favor of Bi-Factor Models in Cognitive Ability Research?: A Comparison of Fit in Correlated Factors, Higher-Order, and Bi-Factor Models via Monte Carlo Simulations. *Journal of Intelligence*, *3*(1), 2–20. https://doi.org/10.3390/jintelligence3010002

Murray, A. L., & Johnson, W. (2013). The limitations of model fit in comparing the bi-factor versus higher-order models of human cognitive ability structure. *Intelligence*, *41*(5), 407–422. https://doi.org/10.1016/j.intell.2013.06.004

Muthén, B. (1984). A general structural equation model with dichotomous, ordered categorical, and continuous latent variable indicators. *Psychometrika*, *49*(1), 115–132. https://doi.org/10.1007/bf02294210

Ringwald, W. R., Forbes, M. K., & Wright, A. G. C. (2023). Meta-analysis of structural evidence for the Hierarchical Taxonomy of Psychopathology (HiTOP) model. *Psychological Medicine*, *53*(2), 533–546. https://doi.org/10.1017/s0033291721001902

Rosseel, Y. (2012). lavaan : An R Package for Structural Equation Modeling. *Journal of Statistical Software*, *48*(2). https://doi.org/10.18637/jss.v048.i02

Shi, D., DiStefano, C., McDaniel, H. L., & Jiang, Z. (2018). Examining Chi-Square Test Statistics Under Conditions of Large Model Size and Ordinal Data. *Structural Equation Modeling: A Multidisciplinary Journal*, *25*(6), 924–945. https://doi.org/10.1080/10705511.2018.1449653

Tucker, L. R., & Lewis, C. (1973). A reliability coefficient for maximum likelihood factor analysis. *Psychometrika*, *38*(1), 1–10. https://doi.org/10.1007/bf02291170

Verona, E., Javdani, S., & Sprague, J. (2011). Comparing Factor Structures of Adolescent Psychopathology. *Psychological Assessment*, *23*(2), 545–551. https://doi.org/10.1037/a0022055

Voorhees, E. E. V., Dennis, P. A., Elbogen, E. B., Clancy, C. P., Hertzberg, M. A., Beckham, J. C., & Calhoun, P. S. (2014). Personality Assessment Inventory internalizing and externalizing structure in veterans with posttraumatic stress disorder: Associations with aggression. *Aggressive Behavior*, *40*(6), 582–592. https://doi.org/10.1002/ab.21554

Waldman, I. D., King, C. D., Poore, H. E., Luningham, J. M., Zinbarg, R. M., Krueger, R. F., … Zald, D. (2022). Recommendations for Adjudicating Among Alternative Structural Models of Psychopathology. *Clinical Psychological Science*, *11*(4), 616–640. https://doi.org/10.1177/21677026221144256

Watts, A. L., Poore, H. E., & Waldman, I. D. (2019). Riskier Tests of the Validity of the Bifactor Model of Psychopathology. *Clinical Psychological Science*, *7*(6), 1285–1303. https://doi.org/10.1177/2167702619855035

eTable 1. *Names and concept codes for initial set of selected diagnoses*

Name of Condition	OMOP ID	SNOMED Code
Abnormal sexual function	4237140	56925008
Acrophobia	4239656	58963008
Adjustment disorder	436677	17226007
Adjustment disorder with anxious mood	436075	47372000
Adjustment disorder with depressed mood	442306	57194009
Adjustment disorder with disturbance of conduct	435799	84984002
Adjustment disorder with mixed anxiety and depressed mood	36684319	782501005
Adjustment disorder with mixed disturbance of emotions AND conduct	436076	66381006
Adjustment disorder with mixed emotional features	433454	55668003
Adjustment disorder with withdrawal	437258	9674006
Agoraphobia	4321835	70691001
Antisocial personality disorder	440988	26665006
Anxiety disorder	442077	197480006
Asperger's disorder	4053178	23560001
Attention deficit hyperactivity disorder	438409	406506008
Autism spectrum disorder	439776	35919005
Autistic disorder	439780	408856003
Avoidant personality disorder	437524	37746008
Bipolar affective disorder, current episode depression	439254	191627008
Bipolar affective disorder, current episode manic	440078	191618007
Bipolar affective disorder, current episode mixed	435226	192362008
Bipolar affective disorder, currently depressed, in full remission	439251	191634005
Bipolar affective disorder, currently depressed, mild	439253	191629006
Bipolar affective disorder, currently depressed, moderate	437528	191630001
Bipolar affective disorder, currently manic, in full remission	439255	191625000
Bipolar affective disorder, currently manic, mild	441834	191620005
Bipolar affective disorder, currently manic, moderate	433992	191621009
Bipolar affective disorder, currently manic, severe, with psychosis	439256	191623007
Bipolar affective disorder, most recent episode mixed	35624745	767633005
Bipolar disorder	436665	13746004
Bipolar disorder in full remission	4220618	41836007
Bipolar disorder in partial remission	436072	5703000
Bipolar disorder in remission	4310821	85248005
Bipolar disorder, most recent episode depression	35624743	767631007
Bipolar disorder, most recent episode manic	35624744	767632000
Bipolar I disorder	432876	371596008
Bipolar I disorder, most recent episode hypomanic	4150985	31446002
Bipolar I disorder, single manic episode	432866	9340000
Bipolar I disorder, single manic episode, in full remission	4148842	3530005
Bipolar I disorder, single manic episode, in remission	4327669	75360000
Bipolar II disorder	4307956	83225003
Bipolar II disorder, most recent episode major depressive	4037669	16295005
Bipolar type I disorder currently in full remission	37109940	723903001
Borderline personality disorder	434626	20010003
Cannabis abuse	434327	37344009
Cannabis dependence	440387	85005007
Cannabis-induced organic mental disorder	4300092	77355000
Chronic depressive personality disorder	40481798	442057004
Claustrophobia	4058397	19887002
Cluster A personality disorder	4043918	16805009
Cluster B personality disorder	4181019	4306003
Cluster C personality disorder	4222618	83890006
Conduct disorder	443617	430909002
Cyclothymia	440696	76105009
Delusional disorder	432590	48500005
Dependent personality disorder	437523	84466009
Depressed bipolar I disorder	441836	49468007
Depressed bipolar I disorder in full remission	435225	22121000
Depressed bipolar I disorder in partial remission	4177651	49512000
Depressed bipolar I disorder in remission	4201739	53607008
Disorders of initiating and maintaining sleep	439708	194437008
Dissociative disorder	434889	44376007
Disturbance in speech	435642	29164008
Dyspareunia	439080	71315007
Dysthymia	433440	78667006
Eating disorder	439002	72366004
Emotionally unstable personality disorder	4103399	191765005

Explosive personality disorder	432611	231527003
Fear of flying	4085062	247853008
Fear of insects	4023785	19512009
Flying phobia	4085680	247854002
Generalized anxiety disorder	434613	21897009
Gilles de la Tourette's syndrome	379782	5158005
Gynephobia	4012108	102930000
Histrionic personality disorder	440369	55341008
Inhibited female orgasm	442749	60103007
Inhibited male orgasm	444268	81903006
Insomnia	436962	193462001
Intellectual disability	40277917	110359009
Intermittent explosive disorder	440989	40987004
Lack or loss of sexual desire	443262	270903007
Major depression, single episode	4282096	36923009
Major depressive disorder	4152280	370143000
Mania	4333677	231494001
Manic bipolar I disorder	4287544	68569003
Manic bipolar I disorder in full remission	436086	30935000
Manic bipolar I disorder in partial remission	442600	63249007
Manic bipolar I disorder in remission	4166701	45479006
Manic disorder, single episode	443237	268619003
Mild bipolar disorder	4028027	13313007
Mild bipolar I disorder, single manic episode	432290	41552001
Mild depression	4149320	310495003
Mild major depression	4336957	87512008
Mild major depression, single episode	4195572	79298009
Mild manic bipolar I disorder	4215917	71984005
Mild recurrent major depression	4228802	40379007
Mixed bipolar affective disorder	439250	191636007
Mixed bipolar affective disorder, in full remission	439245	191643001
Mixed bipolar affective disorder, mild	439249	191638008
Mixed bipolar affective disorder, moderate	439248	191639000
Mixed bipolar affective disorder, severe, with psychosis	439246	191641004
Mixed bipolar I disorder	443906	16506000
Mixed bipolar I disorder in full remission	4009648	111485001
Mixed bipolar I disorder in partial remission	437529	36583000
Mixed bipolar I disorder in remission	433743	35481005
Moderate bipolar disorder	4194222	79584002
Moderate bipolar I disorder, single manic episode	440067	28884001
Moderate depressed bipolar I disorder	4280361	66631006
Moderate major depression	4307111	832007
Moderate major depression, single episode	4049623	15639000
Moderate manic bipolar I disorder	4307804	82998009
Moderate recurrent major depression	4077577	18818009
Narcissistic personality disorder	440080	80711002
Needle phobia	4332995	231501003
Obsessive compulsive personality disorder	443876	1376001
Obsessive-compulsive disorder	440374	191736004
Oppositional defiant disorder	441547	18941000
Orgasm disorder	4221288	82636008
Panic disorder	436074	371631005
Panic disorder with agoraphobia	4147466	35607004
Panic disorder without agoraphobia	4211231	56576003
Paranoid personality disorder	440691	13601005
Personality disorder	441838	33449004
Phobic disorder	4304010	386810004
Posttraumatic stress disorder	436676	47505003
Premature ejaculation	434319	44001008
Psychosexual disorder	436666	56627002
Psychosexual dysfunction	440068	268637002
Psychosexual dysfunction associated with inhibited sexual excitement	44782768	153151000119100
Psychosis and severe depression co-occurrent and due to bipolar affective disorder	35622934	765176007
Psychotic disorder	436073	69322001
Recurrent major depression	4282316	66344007
Schizoaffective disorder, bipolar type	4244078	38368003
Schizoid personality disorder	440085	52954000
Schizotypal personality disorder	434010	31027006
Severe bipolar disorder	4155798	371600003
Severe bipolar disorder with psychotic features	4195158	4441000
Severe bipolar disorder without psychotic features	4200385	53049002

Severe bipolar I disorder	4154283	371599001
Severe bipolar I disorder, single manic episode with psychotic features	4220617	41832009
Severe bipolar I disorder, single manic episode without psychotic features	4030856	14495005
Severe depressed bipolar I disorder	42872413	261000119107
Severe depressed bipolar I disorder with psychotic features	436386	59617007
Severe depressed bipolar I disorder without psychotic features	442570	61403008
Severe major depression	42872722	450714000
Severe major depression, single episode, without psychotic features	441534	76441001
Severe manic bipolar I disorder	43020451	23741000119105
Severe manic bipolar I disorder without psychotic features	443797	162004
Severe mixed bipolar I disorder	42872412	271000119101
Severe mixed bipolar I disorder without psychotic features	372599	46229002
Severe recurrent major depression without psychotic features	435220	36474008
Sexual desire disorder	4186941	46762006
Sleep disorder	435524	39898005
Social phobia	440690	25501002
Substance abuse	4279309	66214007
Tic disorder	381839	568005
Tobacco dependence syndrome	437264	89765005
Zoophobia	4184002	54307006

eTable 2. *Frequencies and diagnostic codes for psychiatric conditions used in analyses*

Name of Condition	N	OMOP ID	SNOMED Code	Source Vocabulary	Source Code
**Acute stress disorder**	7,129	440083	67195008	ICD9CM	308.9
**Adjustment disorder**	6,532	436677	17226007	ICD10CM	F43.20
**Agoraphobia**	181				
Agoraphobia		4321835	70691001	ICD10CM	F40.00
Agoraphobia without history of panic disorder		4189538	61569007	ICD10CM	F40.02
**Alcohol abuse**	12,574	433753	15167005	ICD10CM	F10.180
**Anorexia nervosa**	278	436675	56882008	ICD10CM	F50.00
**Antisocial personality disorder**	461	440988	26665006	ICD10CM	F60.2
**Attention deficit hyperactivity disorder**	4,805	438409	406506008	ICD9CM	314.9
**Attention deficit hyperactivity disorder, combined type**	1,012	4149904	31177006	ICD10CM	F90.2
**Attention deficit hyperactivity disorder, predominantly hyperactive impulsive type**	393	4253962	7461003	ICD10CM	F90.1
**Attention deficit hyperactivity disorder, predominantly inattentive type**	3,133	4149353	35253001	ICD10CM	F90.0
**Autism spectrum disorder**	484				
Autism spectrum disorder		439776	35919005	ICD9CM	299.8
Autistic disorder		439780	408856003	ICD10CM	F84.0
Asperger's disorder		4053178	23560001	ICD10CM	F84.5
**Bipolar disorder**	10,096	436665	13746004	ICD10CM	F31.9
**Borderline personality disorder**	1,935	434626	20010003	ICD10CM	F60.3
**Bulimia nervosa**	375	438407	78004001	ICD9CM	307.51
**Cannabis use**	6,225				
Cannabis abuse		434327	37344009	ICD9CM	305.21
Cannabis dependence		440387	85005007	ICD10CM	F12.20
**Claustrophobia**	1,146	4058397	19887002	ICD10CM	F40.240
**Cocaine abuse**	4,777	432303	78267003	ICD10CM	F14.14
**Conduct disorder**	662	443617	430909002	ICD10CM	F91.8
**Delusional disorder**	1,318	432590	48500005	ICD10CM	F22
**Disturbance in speech**	2,638	435642	29164008	ICD9CM	784.5
**Dysthymia**	11,625				
Dysthymia		433440	78667006	ICD10CM	F34.1
Mild depression		4149320	310495003	NA	NA
**Eating disorder**	2,621	439002	72366004	SNOMED	72366004
**Fear of flying**	223	4085062	247853008	ICD10CM	F40.243
**Generalized anxiety disorder**	19,018	434613	21897009	ICD10CM	F41.1
**Gilles de la Tourette's syndrome**	117	379782	5158005	ICD10CM	F95.2
**Hallucinogen abuse**	129	437245	74851005	ICD10CM	F16.10
**Intellectual disability**	452	40277917	110359009	ICD10CM	F79
**Intermittent explosive disorder**	241	440989	40987004	ICD9CM	312.34
**Major depression**	49,262				
Mild major depression		4336957	87512008	SNOMED	87512008
Mild major depression, single episode		4195572	79298009	ICD9CM	296.21
Mild recurrent major depression		4228802	40379007	ICD10CM	F33.0
Moderate major depression		4307111	832007	SNOMED	832007
Recurrent major depression		4282316	66344007	ICD9CM	296.3
Severe major depression		42872722	450714000	SNOMED	450714000
Severe major depression, single episode, without psychotic features		441534	76441001	ICD9CM	296.23
Severe recurrent major depression without psychotic features		435220	36474008	ICD10CM	F33.2
**Narcissistic personality disorder**	178	440080	80711002	ICD9CM	301.81
**Obsessive compulsive personality disorder**	183	443876	1376001	ICD9CM	301.4
**Obsessive-compulsive disorder**	1,638	440374	191736004	ICD10CM	F42
**Opioid abuse**	4,383	438130	5602001	ICD10CM	F11.10
**Oppositional defiant disorder**	255	441547	18941000	ICD9CM	313.81
**Panic disorder**	8,608				
Panic disorder		436074	371631005	SNOMED	371631005
Panic disorder with agoraphobia		4147466	35607004	ICD9CM	300.21
Panic disorder without agoraphobia		4211231	56576003	ICD10CM	F41.0
**Posttraumatic stress disorder**	10,837	436676	47505003	ICD10CM	F43.10
**Schizoaffective disorder**	2,473	4286201	68890003	ICD10CM	F25.9
**Schizophrenia**	3,154	435783	58214004	ICD10CM	F20.89
**Sedative abuse**	347	442601	64386003	ICD10CM	F13.11
**Sleep disorder (used as an external correlate)**	12,985	435524	39898005	ICD10CM	G47.9
**Social phobia**	947	440690	25501002	ICD10CM	F40.10
**Tic disorder**	228	381839	568005	ICD10CM	F95.9
**Tobacco dependence syndrome**	21,187	437264	89765005	ICD9CM	305.1

eTable 3. *All of Us sample characteristics across race-ethnicity*

	White (N=72,806)	African American (N=24,952)	Asian (N=2,146)	Multiracial (N=2,235)	Hispanic only (N=18,978)	Hispanic (N=21,693)	Non-Hispanic (N=106,270)	Other (N=783)	Not reported (N=6,063)	Total (N=127,963)
**Sex Assigned at Birth**										
Female	44,587 (61.2%)	15,035 (60.3%)	1,328 (61.9%)	1,509 (67.5%)	12,662 (66.7%)	14,452 (66.6%)	63,279 (59.5%)	400 (51.1%)	2,210 (36.5%)	77,731 (60.7%)
Male	27,514 (37.8%)	9,479 (38.0%)	799 (37.2%)	691 (30.9%)	6,129 (32.3%)	7,029 (32.4%)	39,512 (37.2%)	367 (46.9%)	1,562 (25.8%)	46,541 (36.4%)
No matching concept	42 (0.1%)	43 (0.2%)	4 (0.2%)	3 (0.1%)	15 (0.1%)	17 (0.1%)	2,160 (2.0%)	1 (0.1%)	2,069 (34.1%)	2,177 (1.7%)
Not male, not female, prefer not to answer, or skipped	663 (0.9%)	395 (1.6%)	15 (0.7%)	32 (1.4%)	172 (0.9%)	195 (0.9%)	1,319 (1.2%)	15 (1.9%)	222 (3.7%)	1,514 (1.2%)
**Gender**										
Female	44,222 (60.7%)	14,988 (60.1%)	1,311 (61.1%)	1,458 (65.2%)	12,572 (66.2%)	14,328 (66.0%)	62,760 (59.1%)	395 (50.4%)	2,142 (35.3%)	77,088 (60.2%)
Male	27,289 (37.5%)	9,443 (37.8%)	794 (37.0%)	689 (30.8%)	6,067 (32.0%)	6,952 (32.0%)	39,217 (36.9%)	360 (46.0%)	1,527 (25.2%)	46,169 (36.1%)
Not man only, not woman only, prefer not to answer, or skipped	1,295 (1.8%)	521 (2.1%)	41 (1.9%)	88 (3.9%)	339 (1.8%)	413 (1.9%)	4293 (4.0%)	28 (3.6%)	2,394 (39.5%)	4,706 (3.7%)
**Sexual Orientation**										
Heterosexual	64,718 (88.9%)	21,512 (86.2%)	1,881 (87.7%)	1,790 (80.1%)	16,741 (88.2%)	18,929 (87.3%)	91,515 (86.1%)	684 (87.4%)	3,118 (51.4%)	110,444 (86.3%)
All other orientations	8,088 (11.1%)	3,440 (13.8%)	265 (12.3%)	445 (19.9%)	2,237 (11.8%)	2,764 (12.7%)	14,755 (13.9%)	99 (12.6%)	2,945 (48.6%)	17,519 (13.7%)
**Household Income ($/year)**										
Less than 10000	6,916 (9.5%)	8,224 (33.0%)	151 (7.0%)	374 (16.7%)	4,132 (21.8%)	4,686 (21.6%)	15,971 (15.0%)	100 (12.8%)	760 (12.5%)	20,657 (16.1%)
10000 to 25000	8,639 (11.9%)	4,591 (18.4%)	171 (8.0%)	339 (15.2%)	3,029 (16.0%)	3,427 (15.8%)	14,017 (13.2%)	107 (13.7%)	568 (9.4%)	17,444 (13.6%)
25000 to 35000	5,518 (7.6%)	1,823 (7.3%)	104 (4.8%)	172 (7.7%)	1,398 (7.4%)	1,634 (7.5%)	7,700 (7.2%)	44 (5.6%)	275 (4.5%)	9,334 (7.3%)
35000 to 50000	6,614 (9.1%)	1,462 (5.9%)	173 (8.1%)	199 (8.9%)	1,166 (6.1%)	1,389 (6.4%)	8,573 (8.1%)	66 (8.4%)	282 (4.7%)	9,962 (7.8%)
50000 to 75000	9,651 (13.3%)	1,307 (5.2%)	221 (10.3%)	209 (9.4%)	998 (5.3%)	1,267 (5.8%)	11,485 (10.8%)	78 (10.0%)	288 (4.8%)	12,752 (10.0%)
75000 to 100000	7,567 (10.4%)	614 (2.5%)	219 (10.2%)	168 (7.5%)	510 (2.7%)	687 (3.2%)	8,683 (8.2%)	52 (6.6%)	240 (4.0%)	9,370 (7.3%)
100000 to 150000	9,084 (12.5%)	467 (1.9%)	297 (13.8%)	171 (7.7%)	446 (2.4%)	626 (2.9%)	10,113 (9.5%)	57 (7.3%)	217 (3.6%)	10,739 (8.4%)
150000 to 200000	3,967 (5.4%)	148 (0.6%)	145 (6.8%)	75 (3.4%)	133 (0.7%)	213 (1.0%)	4,370 (4.1%)	35 (4.5%)	80 (1.3%)	4,583 (3.6%)
More than 200000	5,254 (7.2%)	139 (0.6%)	230 (10.7%)	119 (5.3%)	166 (0.9%)	245 (1.1%)	5,845 (5.5%)	48 (6.1%)	134 (2.2%)	6,090 (4.8%)
Not reported	9,596 (13.2%)	6,177 (24.8%)	435 (20.3%)	409 (18.3%)	7,000 (36.9%)	7,519 (34.7%)	19,513 (18.4%)	196 (25.0%)	3,219 (53.1%)	27,032 (21.1%)
**Education**										
Less than a high school degree or equivalent	2,791 (3.8%)	3,930 (15.8%)	40 (1.9%)	140 (6.3%)	5,629 (29.7%)	5,894 (27.2%)	7,074 (6.7%)	34 (4.3%)	404 (6.7%)	12,968 (10.1%)
Highest Grade: Twelve Or GED	11,736 (16.1%)	8,275 (33.2%)	150 (7.0%)	398 (17.8%)	5,059 (26.7%)	5,635 (26.0%)	21,030 (19.8%)	137 (17.5%)	910 (15.0%)	26,665 (20.8%)
Highest Grade: College One to Three	20,979 (28.8%)	7,555 (30.3%)	320 (14.9%)	742 (33.2%)	4,632 (24.4%)	5,595 (25.8%)	29,867 (28.1%)	168 (21.5%)	1,066 (17.6%)	35,462 (27.7%)
College graduate or advanced degree	36,566 (50.2%)	4,078 (16.3%)	1,602 (74.7%)	903 (40.4%)	3,087 (16.3%)	3,965 (18.3%)	43,947 (41.4%)	419 (53.5%)	1,257 (20.7%)	47,912 (37.4%)
Not reported	734 (1.0%)	1,114 (4.5%)	34 (1.6%)	52 (2.3%)	571 (3.0%)	604 (2.8%)	4,352 (4.1%)	25 (3.2%)	2,426 (40.0%)	4,956 (3.9%)
**Marital Status**										
Married	35,585 (48.9%)	4,446 (17.8%)	1,054 (49.1%)	661 (29.6%)	6,201 (32.7%)	6,958 (32.1%)	42,587 (40.1%)	336 (42.9%)	1,262 (20.8%)	49,545 (38.7%)
Living With Partner	4,198 (5.8%)	1,460 (5.9%)	111 (5.2%)	207 (9.3%)	1,618 (8.5%)	1,910 (8.8%)	5,938 (5.6%)	34 (4.3%)	220 (3.6%)	7,848 (6.1%)
Divorced	12,266 (16.8%)	4,432 (17.8%)	187 (8.7%)	305 (13.6%)	3,306 (17.4%)	3,709 (17.1%)	17,582 (16.5%)	113 (14.4%)	682 (11.2%)	2,1291 (16.6%)

Never Married	13,581 (18.7%)	10,111 (40.5%)	642 (29.9%)	839 (37.5%)	4,781 (25.2%)	5,766 (26.6%)	25,412 (23.9%)	228 (29.1%)	996 (16.4%)	31,178 (24.4%)
Separated	1,568 (2.2%)	1,657 (6.6%)	32 (1.5%)	68 (3.0%)	1,432 (7.5%)	1,537 (7.1%)	3,402 (3.2%)	19 (2.4%)	163 (2.7%)	4,939 (3.9%)
Widowed	4,647 (6.4%)	1,631 (6.5%)	76 (3.5%)	85 (3.8%)	983 (5.2%)	1,066 (4.9%)	6,659 (6.3%)	31 (4.0%)	272 (4.5%)	7,725 (6.0%)
Not reported	961 (1.3%)	1,215 (4.9%)	44 (2.1%)	70 (3.1%)	657 (3.5%)	747 (3.4%)	4,690 (4.4%)	22 (2.8%)	2,468 (40.7%)	5,437 (4.2%)

eTable 4. *Fit and alternative indices for confirmatory and exploratory models*

							Alternative Indices			Errors
**Model**	**χ^2**	**df**	**CFI**	**TLI**	**RMSEA**	**SRMR**	**FL**	**SD**	**SE**	
**1.** General factor	43,769	702	.807	.797	0.022	0.13	.493	.167	.014	
Correlated factors (I=Internalizing, E=Externalizing, T=Thought problems, S=Substance use, N=Neurodevelopmental disorders, F=Fear, D = Distress)										
**2.** I, E, T	24,669	591	.890	.883	.018	.123	.556	.182	.015	
**3.** I, E, T, S	18,888	588	.916	.910	.016	.115	.631	.194	.015	
**4.** I, E, T, N	25,128	696	.891	.884	.017	.120	.567	.170	.016	
**5.** I, E, T, S, N	19,187	692	.917	.911	.014	.108	.604	.177	.016	Yes
**6.** F, D, E, T	24,535	588	.891	.883	.018	.123	.572	.182	.015	
**7.** F, D, E, T, S	18,750	584	.917	.910	.016	.114	.644	.192	.016	
**8.** F, D, E, T, N	24,895	690	.892	.884	.017	.119	.587	.278	.020	
**9.** F, D, E, T, S, N	19,050	687	.918	.911	.014	.107	.613	.175	.016	Yes
Tests of the placement of ambiguously classified disorders										
ADHD and its three subtypes										
Placement in model 9: ADHD-HI & ADHD-C on E; ADHD-IN on N										
**9.1.** Six correlated factors (ADHD on E only)	17,449	614	.923	.917	.015	.101	.655	.181	.016	
**9.2.** Six correlated factors (ADHD on N only)	17,329	614	.924	.917	.015	.101	.615	.182	.016	
**9.3.** Six correlated factors (ADHD on E and N)	17,293	613	.924	.917	.015	.101	.622	.192	.017	
**9.4.** Six correlated factors (ADHD-HI and ADHD-C on E; ADHD-IN and ADHD-C on N)	18,699	686	.919	.913	.014	.105	.619	.215	.019	
**9.5.** Six correlated factors (ADHD-HI on E; ADHD-IN and ADHD-C on N. Correlated residuals between ADHD-HI and ADHD-IN as well as ADHD-HI and ADHD-C)	18,299	685	.921	.915	.014	.102	.616	.176	.017	
**9.6.** Six correlated factors (ADHD-C on E; ADHD-HI and ADHD-IN on N. Correlated residuals between ADHD-C and ADHD-HI as well as ADHD-C and ADHD-IN)	18,322	685	.921	.915	.014	.103	.622	.177	.017	
Eating Disorder (ED), Anorexia Nervosa (AN), and Bulimia Nervosa (BN)										
Placement in model 9.5: ED on T										
**9.5.1.** Six correlated factors (ED on D only)	17,550	685	.925	.918	.014	.101	.614	.174	.017	
**9.5.2.** Six correlated factors (ED on D and T)	17,530	684	.925	.918	.014	.101	.614	.201	.017	
**9.5.3.** Six correlated factors (AN on D and T; BN on D and E)	17,880	721	.924	.917	.014	.103	.613	.211	.019	
**9.5.4.** Six correlated factors (AN on D; BN on D and E)	17,880	723	.924	.918	.014	.103	.614	.168	.017	
**9.5.5.** Six correlated factors (AN on T; BN on E)	18,299	723	.922	.916	.014	.107	.615	.166	.017	
Obsessive-Compulsive Disorder (OCD) & Obsessive-Compulsive Personality Disorder (OCPD)										
Placement in model 9.5: OCD & OCPD on T										
**9.5.6.** Six correlated factors (OCD and OCPD on F)	16,740	685	.928	.922	.014	.097	.654	.178	.017	
**9.5.7.** Six correlated factors (OCD and OCPD on N)	16,765	685	.928	.922	.014	.098	.611	.182	.016	
**9.5.8.** Six correlated factors (OCD and OCPD on F and N)	16,418	683	.930	.924	.013	.096	.606	.183	.018	
**9.5.9.** Six correlated factors (OCD and OCPD on D)	16,739	685	.928	.922	.014	.097	.637	.177	.017	
Borderline Personality Disorder (BPD)										
Placement in model 9.5: BPD on T										
**9.5.10.** Six correlated factors (BPD on E)	17,244	685	.926	.920	.014	.101	.611	.176	.016	
**9.5.11.** Six correlated factors (BPD on D)	17,775	685	.924	.917	.014	.103	.619	.179	.017	
**9.5.12.** Six correlated factors (BPD on D and E)	17,051	684	.927	.921	.014	.101	.617	.172	.017	
**9.5.13.** Six correlated factors (BPD on D and T)	16,773	684	.928	.922	.014	.102	.615	.177	.017	
**9.5.14.** Six correlated factors (BPD on E and T)	17,238	684	.926	.920	.014	.101	.610	.188	.017	
Tic and Tourette's (TS)										
Placement in model 9.5: Tic and TS on N										
**9.5.15.** Six correlated factors (TIC and TS on D)	18,548	685	.920	.914	.014	.105	.619	.185	.016	
**9.5.16.** Six correlated factors (TIC and TS on N and D)	18,275	683	.921	.915	.014	.103	.634	.237	.019	
Antisocial personality disorder (ASPD)										
Placement in model 9.5: ASPD on S										
**9.5.17.** Six correlated factors (ASPD on E and S)	17,904	684	.923	.917	.014	.101	.614	.172	.017	
Additional Correlated Residuals (~~)										
**9.5.18.** Six correlated factors (SCZ ~~ SCZAFF)	16,761	684	.928	.922	.014	.100	.614	.172	.017	
**9.5.19.** Six correlated factors (OCD ~~ OCPD)	17,929	684	.923	.916	.014	.100	.617	.178	.017	
**9.5.20.** Six correlated factors (MDD ~~ dysthymia)	17,863	684	.923	.917	.014	.102	.615	.177	.017	
**9.5.21.** Six correlated factors (TIC ~~ TS)	18,131	684	.922	.916	.014	.100	.615	.180	.017	
**9.5.22.** Six correlated factors (CD ~~ ODD)	18,172	684	.922	.915	.014	.100	.607	.172	.017	Yes
**9.5.23.** Six correlated factors (OCD ~~ TS)	18,243	684	.922	.915	.014	.102	.616	.176	.017	
**9.5.24.** Six correlated factors (OCD ~~ TIC)	18,254	684	.921	.915	.014	.102	.616	.176	.017	

**9.5.25.** Six correlated factors (PANIC ~~ AGOR)	18,270	684	.921	.915	.014	.102	.616	.177	.017
**9.5.26.** Six correlated factors (SCZ ~~ SCZAFF; OCD ~~ OCPD; MDD ~~ dysthymia)	15,962	682	.932	.926	.013	.098	.617	.174	.017
Models Based on Modification Indices									
PTSD *- Previous placement in Model 9.5.26: PTSD on D*									
**9.5.26.1.** Six correlated factors (PTSD on T)	14,537	682	.938	.933	.013	.097	.616	.169	.017
**9.5.26.2.** Six correlated factors (PTSD on T and D)	13,428	681	.943	.938	.012	.097	.614	.175	.017
OCD *- Previous placement in Model 9.5.26: OCD on D*									
**9.5.26.2.1.** Six correlated factors (OCD on T and D)	12,147	680	.949	.944	.011	.094	.611	.188	.017
BPD *- Previous placement in Model 9.5.26: BPD on T*									
**9.5.26.2.1.1.** Six correlated factors (BPD on T and D)	10,858	679	.954	.950	.011	.093	.605	.185	.017
ED *- Previous placement in Model 9.5.26: ED on T*									
**9.5.26.2.1.1.1.** Six correlated factors (ED on T and D)	9,469	678	.961	.957	.010	.091	.598	.202	.017

Note. CFI – comparative fit index, TLI – tucker-lewis index, RMSEA - root mean square error of approximation, SRMR – standardized root mean squared residual, FL – median factor loading, SD – standard deviation of loadings, SE – mean standard error. ADHD-HI – ADHD hyperactive-impulsive subtype, ADHD-IN – ADHD inattentive subtype, ADHD-C = ADHD combined subtype, SCZ – schizophrenia, SCZAFF – schizoaffective, CD – conduct disorder, ODD – oppositional defiant disorder, PANIC – panic disorder, AGOR – agoraphobia. Model in bold is our preferred best-fitting model.

eTable 5. *Alternative indices for fear and distress diagnoses across confirmatory models*

	Alternative Indices Fear Diagnoses			Alternative Indices Distress Diagnoses		
**Model**	**FL**	**SD**	**SE**	**FL**	**SD**	**SE**
**2.** I, E, T	.525	.270	.020	.464	.154	.013
**3.** I, E, T, S	.530	.267	.020	.468	.150	.013
**4.** I, E, T, N	.533	.270	.020	.549	.124	.015
**5.** I, E, T, S, N	.535	.268	.020	.545	.121	.015
**6.** F, D, E, T	.580	.301	.022	.461	.153	.013
**7.** F, D, E, T, S	.588	.298	.022	.465	.149	.022
**8.** F, D, E, T, N	.588	.300	.022	.463	.434	.020
**9.** F, D, E, T, S, N	.591	.298	.022	.547	.140	.007

Note. F = fear, D = distress, E = externalizing, S = substance use, T = thought problems, N = neurodevelopmental disorders. FL = median factor loading, SD = standard deviation of loadings, SE = mean standard error.

eTable 6. *Fit and alternative indices for bifactor and modified bifactor versions of best fitting models*

Model	χ^2	df	CFI	TLI	RMSEA	SRMR	Alternative Indices FL	SD	SE	Errors
**Bifactor (Orthogonal specific factors)**										
**2.** I, E, T	12,161	558	.947	.940	.013	.097	.298	.369	.018	
**3.** I, E, T, S	12,169	558	.947	.940	.013	.094	.429	.314	.019	
**4.** I, E, T, N	14,789	663	.937	.929	.013	.098	.363	.343	.020	
**5.** I, E, T, S, N	15,459	663	.934	.926	.013	.099	.457	.313	.021	
**6.** F, D, E, T	17,249	558	.924	.914	.015	.101	.305	.359	.018	
**7.** F, D, E, T, S	16,993	558	.925	.915	.015	.099	.389	.317	.019	
**8.** F, D, E, T, N	19,486	661	.916	.906	.015	.100	.359	.322	.022	
**9.** F, D, E, T, S, N	20,171	663	.913	.903	.015	.102	.421	.301	.031	
**9.5.** Six correlated factors (ADHD-HI on E; ADHD-IN and ADHD-C on N)	17,354	661	.925	.916	.014	.097	.398	.295	.021	
**9.5.26.** Six correlated factors (9.5 + SCZ ~~ SCZAFF; OCD ~~ OCPD; MDD ~~ dysthymia)	17,069	658	.927	.917	.014	.095	.416	.271	.029	
**9.5.26.2.1.1.1.** Six correlated factors (9.5.27 + PTSD on T and D; OCD on T and D; BPD on T and D; ED on D and T)	16,360	654	.930	.92	.014	.095	.379	.280	.024	Yes
**Modified Bifactor (Correlated specific factors)**										
**2.** I, E, T	7,005	555	.971	.967	.010	.081	.397	.314	.018	
**3.** I, E, T, S	5,756	552	.976	.973	.009	.076	.420	.279	.019	
**4.** I, E, T, N	7,466	657	.970	.966	.009	.080	.425	.298	.019	
**5.** I, E, T, S, N	6,351	653	.975	.971	.008	.077	.419	.276	.020	Yes
**6.** F, D, E, T	6,922	552	.971	.967	.009	.080	.401	.315	.018	
**7.** F, D, E, T, S	5,681	548	.977	.973	.009	.075	.412	.280	.019	
**8.** F, D, E, T, N	7,375	651	.970	.966	.009	.080	.415	.304	.019	
**9.** F, D, E, T, S, N	6,286	648	.975	.971	.008	.077	.420	.276	.020	Yes
**9.5.** Six correlated factors (ADHD-HI on E; ADHD-IN and ADHD-C on N)	5,767	646	.977	.974	.008	.072	.402	.257	.021	
**9.5.26.** Six correlated factors (9.5 + SCZ ~~ SCZAFF; OCD ~~ OCPD; MDD ~~ dysthymia)	5,095	643	.980	.977	.007	.070	.400	.250	.022	
**9.5.26.2.1.1.1.** Six correlated factors (9.5.27 + PTSD on T and D; OCD on T and D; BPD on T and D; ED on D and T)	4,912	639	.981	.978	.007	.069	.395	.396	.051	

Note. CFI – comparative fit index, TLI – tucker-lewis index, RMSEA - root mean square error of approximation, SRMR – standardized root mean squared residual, FL – median factor loading, SD – standard deviation of loadings, SE – mean standard error. ADHD-HI – ADHD hyperactive-impulsive subtype, ADHD-IN – ADHD inattentive subtype, ADHD-C = ADHD combined subtype, SCZ – schizophrenia, SCZAFF – schizoaffective, CD – conduct disorder, MDD – major depressive disorder, OCD – obsessive-compulsive disorder, BPD – borderline personality disorder, ED – eating disorder.

eTable 7. *Comparisons among factors in Model 9.5 in their alternative indices*

Index / Factor	Mean FLs	Median FLs	SD of FLs	Mean SEs of FLs	Median SEs of FLs
**Externalizing**	.688	.700	.110	.027	.028
**Substance Use**	.692	.693	.096	.012	.007
**Thought Problems**	.641	.670	.169	.012	.011
**Fear**	.431	.591	.297	.022	.018
**Distress**	.520	.547	.014	.007	.006
**NDD**	.587	.616	.135	.026	.023

eTable 8. *Split-half replication of all models*

							Alternative Indices		
**Model**	**χ²**	**df**	**CFI**	**TLI**	**RMSEA**	**SRMR**	**FL**	**SD**	**SE**
**1.** General factor	43,769	702	0.807	0.797	0.022	0.127	.493	.17	.01
**a. First random half**	22,695	702	0.806	0.795	0.022	0.13	.468	.160	.02
**b. Second random half**	22,115	702	0.808	0.798	0.022	0.13	.505	.165	.02
**2.** I, E, T	24,669	591	0.89	0.883	0.018	0.123	0.56	0.18	0.01
**a. First random half**	12,381	591	0.893	0.886	0.018	0.124	0.54	0.17	0.02
**b. Second random half**	13,169	591	0.885	0.877	0.018	0.13	0.57	0.18	0.02
**3.** I, E, T, S	18,888	588	0.916	0.91	0.016	0.115	0.63	0.19	0.02
**a. First random half**	9,636	588	0.918	0.912	0.016	0.116	0.64	0.18	0.02
**b. Second random half**	10,159	588	0.912	0.906	0.016	0.121	0.64	0.19	0.02
**4.** I, E, T, N	25,128	696	0.891	0.884	0.017	0.12	0.57	0.17	0.02
**a. First random half**	12,774	696	0.893	0.886	0.016	0.123	0.55	0.16	0.02
**b. Second random half**	13,398	696	0.886	0.879	0.017	0.125	0.58	0.17	0.02
**5.** I, E, T, S, N	19,187	692	0.917	0.911	0.014	0.108	0.6	0.18	0.02
**a. First random half**	10,035	692	0.917	0.912	0.015	0.11	0.59	0.17	0.02
**b. Second random half**	10,327	692	0.914	0.908	0.015	0.113	0.62	0.18	0.02
**6.** F, D, E, T	24,535	588	0.891	0.883	0.018	0.123	0.57	0.18	0.02
**a. First random half**	12,320	588	0.894	0.886	0.018	0.124	0.54	0.17	0.02
**b. Second random half**	13,092	588	0.886	0.877	0.018	0.129	0.57	0.18	0.02
**7.** F, D, E, T, S	18,750	584	0.917	0.91	0.016	0.114	0.64	0.19	0.02
**a. First random half**	9,571	584	0.919	0.912	0.016	0.115	0.65	0.18	0.02
**b. Second random half**	9,571	584	0.919	0.912	0.016	0.115	0.65	0.18	0.02
**8.** F, D, E, T, N	24,895	690	0.892	0.884	0.017	0.119	0.59	0.28	0.02
**a. First random half**	12,695	690	0.894	0.886	0.016	0.122	0.55	0.24	0.03
**b. Second random half**	13,272	690	0.887	0.879	0.017	0.124	0.58	0.28	0.03
**9.** F, D, E, T, S, N	19,050	687	0.918	0.911	0.014	0.107	0.61	0.17	0.02
**a. First random half**	9,972	687	0.918	0.911	0.015	0.109	0.62	0.17	0.02
**b. Second random half**	10,240	687	0.914	0.908	0.015	0.112	0.62	0.17	0.02
**9.1.** Six correlated factors (ADHD on E only)	17,449	614	0.923	0.917	0.015	0.101	0.65	0.18	0.02
**a. First random half**	9,113	614	0.923	0.917	0.015	0.104	0.61	0.17	0.02
**b. Second random half**	9,275	614	0.921	0.914	0.015	0.106	0.65	0.18	0.02
**9.2.** Six correlated factors (ADHD on N only)	17,329	614	0.924	0.917	0.015	0.101	0.61	0.18	0.02
**a. First random half**	9,053	614	0.924	0.918	0.015	0.104	0.6	0.17	0.02
**b. Second random half**	9,187	614	0.921	0.915	0.015	0.105	0.61	0.18	0.02
**9.3.** Six correlated factors (ADHD on E and N)	17,293	613	0.924	0.917	0.015	0.101	0.62	0.19	0.02
**a. First random half**	9,036	613	0.924	0.918	0.015	0.104	0.6	0.19	0.02
**b. Second random half**	9,175	613	0.922	0.915	0.015	0.105	0.62	0.19	0.02
**9.4.** Six correlated factors (Hyp-Imp and Combined on E; Inattentive and Combined on N)	18,699	686	0.919	0.913	0.014	0.105	0.62	0.22	0.02
**a. First random half**	9,821	686	0.919	0.913	0.014	0.109	0.64	0.21	0.03
**b. Second random half**	10,009	686	0.917	0.91	0.015	0.109	0.62	0.22	0.03
**9.5.** Six correlated factors (Hyp-Imp on E; Inattentive and COMB on N. Correlated residuals between Hyp-Imp and Inattentive as well as Hyp-Imp and Combined)	18,299	685	.921	.915	.014	.1	.616	.176	.02
**a. First random half**	9,579	685	.921	.915	.014	.107	.640	.170	.02
**b. Second random half**	9,783	685	.919	.912	.014	.107	.640	.180	.02
**9.6.** Six correlated factors (Combined on E; Hyp-Imp and Inattentive on N. Correlated residuals between Combined and Hyp-Imp as well as Combined and Inattentive)	18,322	685	0.921	0.915	0.014	0.103	0.62	0.18	0.02
**a. First random half**	9,562	685	0.921	0.915	0.014	0.105	0.61	0.17	0.02
**b. Second random half**	9,868	685	0.918	0.911	0.014	0.108	0.64	0.18	0.02
**9.5.1.** Six correlated factors (ED on D only)	17,550	685	.925	.918	.014	.1	.614	.174	.02
**a. First random half**	9,176	685	.925	.919	.014	.106	.640	.170	.02
**b. Second random half**	9,437	685	.922	.915	.014	.106	.640	.170	.02
**9.5.2.** Six correlated factors (ED on D and T)	17,530	684	.925	.918	.014	.1	.614	.201	.02
**a. First random half**	9,165	684	.925	.919	.014	.106	.620	.190	.02
**b. Second random half**	9,428	684	.922	.915	.014	.106	.630	.200	.02
**9.5.3.** Six correlated factors (AN on D and T; BN on D and E)	17,880	721	.924	.917	.014	.1	.613	.211	.02
**a. First random half**	9,291	721	.924	.918	.014	.106	.590	.210	.03
**b. Second random half**	9,685	721	.920	.914	.014	.108	.620	.210	.03
**9.5.4.** Six correlated factors (AN on D; BN on D and E)	17,880	723	.924	.918	.014	.1	.614	.168	.02
**a. First random half**	9,293	723	.924	.918	.014	.107	.620	.160	.02

**b. Second random half**	9,686	723	.920	.914	.014	.108	.630	.170	.02
**9.5.5.** Six correlated factors (AN on T; BN on E)	18,299	723	.922	.916	.014	.11	.615	.166	.02
**a. First random half**	9,559	723	.922	.916	.014	.109	.610	.160	.02
**b. Second random half**	9,891	723	.918	.912	.014	.112	.620	.170	.02
**9.5.6.** Six correlated factors (OCD and OCPD on F)	16,740	685	.928	.922	.014	.1	.654	.178	.02
**a. First random half**	8,821	685	.928	.922	.014	.102	.650	.170	.02
**b. Second random half**	8,988	685	.926	.920	.014	.101	.640	.180	.02
**9.5.7.** Six correlated factors (OCD and OCPD on N)	16,765	685	.928	.922	.014	.1	.611	.182	.02
**a. First random half**	8,842	685	.928	.922	.014	.101	.640	.180	.02
**b. Second random half**	9,030	685	.925	.919	.014	.101	.640	.180	.02
**9.5.8.** Six correlated factors (OCD and OCPD on F and N)	16,418	683	.930	.924	.013	.1	.606	.183	.02
**a. First random half**	8,656	683	.929	.924	.014	.100	.600	.180	.02
**b. Second random half**	8,858	683	.927	.921	.014	.100	.620	.190	.02
**9.5.9.** Six correlated factors (OCD and OCPD on D)	16,739	685	.928	.922	.014	.1	.637	.177	.02
**a. First random half**	8,830	685	.928	.922	.014	.101	.640	.170	.02
**b. Second random half**	8,972	685	.926	.920	.014	.101	.640	.180	.02
**9.5.10.** Six correlated factors (BPD on E)	17,244	685	.926	.920	.014	.1	.611	.176	.02
**a. First random half**	9,062	685	.926	.920	.014	.106	.600	.170	.02
**b. Second random half**	9,210	685	.924	.917	.014	.106	.610	.180	.02
**9.5.11.** Six correlated factors (BPD on D)	17,775	685	.924	.917	.014	.1	.619	.179	.02
**a. First random half**	9,367	685	.923	.917	.014	.107	.640	.170	.02
**b. Second random half**	9,478	685	.921	.915	.014	.107	.640	.180	.02
**9.5.12.** Six correlated factors (BPD on D and E)	17,051	684	.927	.921	.014	.1	.617	.172	.02
**a. First random half**	8,974	684	.927	.921	.014	.106	.600	.170	.02
**b. Second random half**	9,120	684	.924	.918	.014	.106	.620	.170	.02
**9.5.13.** Six correlated factors (BPD on D and T)	16,773	684	.928	.922	.014	.1	.615	.177	.02
**a. First random half**	8,819	684	.928	.922	.014	.106	.610	.170	.02
**b. Second random half**	9,022	684	.925	.919	.014	.106	.630	.180	.02
**9.5.14.** Six correlated factors (BPD on E and T)	17,238	684	.926	.920	.014	.1	.61	.188	.02
**a. First random half**	9,056	684	.926	.920	.014	.106	.600	.180	.02
**b. Second random half**	9,210	684	.924	.917	.014	.106	.610	.200	.02
**9.5.15.** Six correlated factors (Tic and TS on D)	18,548	685	.920	.914	.014	.11	.619	.185	.02
**a. First random half**	9,615	685	.921	.915	.014	.108	.640	.170	.02
**b. Second random half**	9,916	685	.917	.911	.015	.109	.630	.180	.02
**9.5.16.** Six correlated factors (Tic and TS on N and D)	18,275	683	.921	.915	.014	.1	.634	.237	.02
**a. First random half**	9,565	683	.921	.915	.014	.107	.620	.200	.03
**b. Second random half**	9,774	683	.919	.912	.014	.107	.630	.230	.03
**9.5.17.** Six correlated factors (ASPD on E and S)	17,904	684	.923	.917	.014	.1	.614	.172	.02
**a. First random half**	9,392	684	.923	.917	.014	.106	.600	.160	.02
**b. Second random half**	9,602	684	.920	.914	.014	.106	.630	.170	.02
**9.5.26.** Six correlated factors (SCZ ~~ SCZAFF; OCD ~~ OCPD; MDD ~~ Dysthymia)	15,962	682	.932	.926	.013	.1	.617	.174	.02
**a. First random half**	8,431	682	.931	.926	.013	.102	.630	.170	.02
**b. Second random half**	8,588	682	.929	.923	.013	.103	.630	.170	.02
**9.5.26.1.** Six correlated factors (PTSD on T)	14,537	682	.938	.933	.013	.1	.616	.169	.02
**a. First random half**	7,882	682	.936	.931	.013	.102	.620	.160	.02
**b. Second random half**	7,717	682	.937	.932	.013	.102	.630	.170	.02
**9.5.26.2.** Six correlated factors (PTSD on T and D)	13,428	681	.943	.938	.012	.1	.614	.175	.02
**a. First random half**	7,203	681	.942	.937	.012	.102	.610	.170	.02
**b. Second random half**	7,277	681	.941	.936	.012	.102	.620	.180	.02
**9.5.26.2.1.** Six correlated factors (OCD on T and D)	12,147	680	.949	.944	.011	.09	.611	.188	.02
**a. First random half**	6,609	680	.948	.943	.012	.100	.600	.180	.02
**b. Second random half**	6,595	680	.947	.942	.012	.099	.630	.190	.02
**9.5.26.2.1.1** Six correlated factors (BPD on T and D)	10,858	679	.954	.950	.011	.09	.605	.185	.02
**a. First random half**	5,963	679	.953	.949	.011	.098	.600	.180	.02
**b. Second random half**	5,957	679	.953	.948	.011	.098	.610	.190	.02
**9.5.26.2.1.1.1.** Six correlated factors (ED on T and D)	9,469	678	.961	.957	.01	.09	.598	.202	.02
**a. First random half**	5,261	678	.959	.956	.010	.097	.600	.190	.02
**b. Second random half**	5,265	678	.959	.955	.010	.096	.590	.200	.02

Note. CFI – comparative fit index, TLI – tucker-lewis index, RMSEA - root mean square error of approximation, SRMR – standardized root mean squared residual, FL – median factor loading, SD – standard deviation of loadings, SE – mean standard error. SCZ – schizophrenia, SCZAFF – schizoaffective, CD – conduct disorder, ODD – oppositional defiant disorder, PANIC – panic disorder, AGOR – agoraphobia. First half N = 63,981, second half N = 63,982.

eTable 9. *Split-half replication of exploratory bifactor models*

							Alternative Indices		
**Model**	**χ²**	**df**	**CFI**	**TLI**	**RMSEA**	**SRMR**	**FL**	**SD**	**SE**
**2.** I, E, T	12160.766	558	0.947	0.94	0.013	0.097	0.3	0.37	0.02
**a. First Random Half**	4254.175	552	0.966	0.962	0.01	0.088	0.59	0.18	
**b. Second Random Half**	3862.891	552	0.97	0.965	0.01	0.085	0.35	0.41	
**3.** I, E, T, S	12168.579	558	0.947	0.94	0.013	0.094	0.43	0.31	0.02
**a. First Random Half**	3170.741	548	0.976	0.973	0.009	0.081	0.39	0.42	
**b. Second Random Half**	3327.887	548	0.975	0.971	0.009	0.081	0.38	0.37	
**4.** I, E, T, N	14789.321	663	0.937	0.929	0.013	0.098	0.36	0.34	0.02
**a. First Random Half**	4614.839	653	0.965	0.96	0.01	0.088	0.53	0.18	
**b. Second Random Half**	4196.918	653	0.968	0.964	0.009	0.083	0.35	0.41	
**5.** I, E, T, S, N	15459.462	663	0.934	0.926	0.013	0.099	0.46	0.31	0.03
**a. First Random Half**	3578.646	648	0.974	0.97	0.008	0.082	0.39	0.49	
**b. Second Random Half**	3705.628	648	0.973	0.969	0.009	0.082	0.42	0.38	
**6.** F, D, E, T	17248.785	558	0.924	0.914	0.015	0.101	0.3	0.36	0.02
**a. First Random Half**	3871.018	548	0.97	0.965	0.01	0.087	0.34	0.49	
**b. Second Random Half**	3811.332	548	0.97	0.966	0.01	0.085	0.34	0.41	
**7.** F, D, E, T, S	16993.299	558	0.925	0.915	0.015	0.099	0.39	0.32	0.02
**a. First Random Half**	3138.617	543	0.977	0.973	0.009	0.081	0.38	0.41	
**b. Second Random Half**	3279.643	543	0.975	0.971	0.009	0.081	0.38	0.36	
**8.** F, D, E, T, N	19485.974	661	0.916	0.906	0.015	0.1	0.36	0.32	0.03
**a. First Random Half**	4172.646	646	0.969	0.964	0.009	0.086	0.32	0.56	
**b. Second Random Half**	4144.169	646	0.969	0.964	0.009	0.083	0.39	0.47	
**9.** F, D, E, T, S, N	20171.114	663	0.913	0.903	0.015	0.102	0.42	0.3	0.03
**a. First Random Half**	3549.477	642	0.974	0.97	0.008	0.082	0.45	0.48	
**b. Second Random Half**	3683.517	642	0.973	0.969	0.009	0.08	0.55	0.87	
**9.5.** Six correlated factors (Hyp-Imp on E; Inattentive and Combined on N)	17354.233	661	0.925	0.916	0.014	0.097	0.4	0.29	0.03
**a. First Random Half**	3579.357	640	0.974	0.97	0.008	0.082	0.62	0.2	
**b. Second Random Half**	3462.609	640	0.975	0.971	0.008	0.078	0.6	0.22	
**9.5.26.** Six correlated factors (SCZ ~~ SCZAFF; OCD ~~ OCPD; MDD ~~ Dysthymia)	17068.688	658	0.927	0.917	0.014	0.095	0.42	0.27	0.03
**a. First random half**	3133.882	637	0.978	0.974	0.008	0.081	0.62	0.2	
**b. Second random half**	2982.571	637	0.979	0.976	0.008	0.077	0.58	0.22	
**9.5.26.2.1.1.1.** Six correlated factors (ED on T and D)	16359.642	654	0.93	0.92	0.014	0.095	0.38	0.28	0.03
**a. First random half**	2991.875	633	0.979	0.976	0.008	0.079	0.52	0.22	
**b. Second random half**	2915.415	633	0.98	0.976	0.008	0.076	0.55	0.24	

Note. CFI – comparative fit index, TLI – tucker-lewis index, RMSEA - root mean square error of approximation, SRMR – standardized root mean squared residual, FL – median factor loading, SD – standard deviation of loadings, SE – mean standard error. SCZ – schizophrenia, SCZAFF – schizoaffective, CD – conduct disorder, ODD – oppositional defiant disorder, PANIC – panic disorder, AGOR – agoraphobia. First half N = 63,981, second half N = 63,982.

eTable 10. *Split-half replication of exploratory modified bifactor models*

							Alternative Indices		
**Model**	**χ²**	**df**	**CFI**	**TLI**	**RMSEA**	**SRMR**	**FL**	**SD**	**SE**
**2.** I, E, T	7004.995	555	.971	.967	.01	.081	.31	0.37	0.02
**a. First Random Half**	3905.403	555	.97	.966	.01	.088	.28	0.37	0.03
**b. Second Random Half**	3862.891	555	.97	.966	.01	.085	.33	0.36	0.03
**3.** I, E, T, S	5756.216	552	.976	.973	.009	.076	.38	0.33	0.02
**a. First Random Half**	3170.741	552	.976	.973	.009	.081	.36	0.33	0.03
**b. Second Random Half**	3327.887	552	.975	.971	.009	.081	.38	0.34	0.03
**4.** I, E, T, N	7466.448	657	.97	.966	.009	.08	.35	0.35	0.02
**a. First Random Half**	4614.839	657	.965	.961	.01	.088	.51	0.18	0.03
**b. Second Random Half**	4196.918	657	.968	.964	.009	.083	.35	0.35	0.03
**5.** I, E, T, S, N	6350.994	653	.975	.971	.008	.077	.43	0.33	0.02
**a. First Random Half**	3842.144	653	.972	.968	.009	.085	.59	0.15	0.03
**b. Second Random Half**	3705.628	653	.973	.969	.009	.082	.41	0.33	0.03
**6.** F, D, E, T	6922.238	552	.971	.967	.009	.08	.32	0.37	0.02
**a. First Random Half**	3871.018	552	.97	.966	.01	.087	.29	0.38	0.03
**b. Second Random Half**	3811.332	552	.97	.966	.01	.085	.33	0.37	0.03
**7.** F, D, E, T, S	5680.54	548	.977	.973	.009	.075	.39	0.34	0.02
**a. First Random Half**	3138.617	548	.977	.973	.009	.081	.36	0.34	0.03
**b. Second Random Half**	3279.643	548	.975	.971	.009	.081	.38	0.34	0.03
**8.** F, D, E, T, N	7374.768	651	.97	.966	.009	.08	0.32	0.36	0.02
**a. First Random Half**	4568.608	651	.965	.961	.01	.087	0.5	0.22	0.03
**b. Second Random Half**	4394.246	651	.966	.962	.009	.082	0.51	0.26	0.03
**9.** F, D, E, T, S, N	6285.615	648	.975	.971	.008	.077	0.42	0.33	0.02
**a. First Random Half**	3795.468	648	.972	.968	.009	.085	0.61	0.15	0.03
**b. Second Random Half**	3683.517	648	.973	.969	.009	.08	0.57	0.16	0.03
**9.5.** Six correlated factors (Hyp-Imp on E; Inattentive and Combined on N)	5766.904	646	.977	.974	.008	.072	0.43	0.31	0.02
**a. First Random Half**	3579.357	646	.974	.97	.008	.082	0.58	0.19	0.03
**b. Second Random Half**	3462.609	646	.975	.971	.008	.078	0.6	0.21	0.03
**9.5.26.** Six correlated factors (SCZ ~~ SCZAFF; OCD ~~ OCPD; MDD ~~ Dysthymia)	5094.605	643	.98	.977	.007	.07	0.4	0.3	0.02
**a. First random half**	3133.882	643	.978	.975	.008	.081	0.58	0.18	0.03
**b. Second random half**	2982.571	643	.979	.976	.008	.077	0.58	0.2	0.03
**9.5.26.2.1.1.1.** Six correlated factors (ED on T and D)	4912.169	639	.981	.978	.007	.069	0.4	0.51	0.08
**a. First random half**	2991.875	639	.979	.976	.008	.079	0.5	0.2	0.03
**b. Second random half**	2915.415	639	.98	.976	.007	.076	0.5	0.22	0.03

Note. CFI – comparative fit index, TLI – tucker-lewis index, RMSEA - root mean square error of approximation, SRMR – standardized root mean squared residual, FL – median factor loading, SD – standard deviation of loadings, SE – mean standard error. SCZ – schizophrenia, SCZAFF – schizoaffective, CD – conduct disorder, ODD – oppositional defiant disorder, PANIC – panic disorder, AGOR – agoraphobia. First half N = 63,981, second half N = 63,982.

eTable 11. *External correlates of the higher-order psychopathology dimensions*

	Externalizing Beta [95% CI]	Substance Use Beta [95% CI]	Thought Problems Beta [95% CI]	Fear Beta [95% CI]	Distress Beta [95% CI]	NDD Beta [95% CI]
**Sex**	.204 [.153 - .255]	.559 [.540 - .578]	.079 [.057 - .102]	-.348 [-.379 - -.317]	-.390 [-.408 - -.372]	.056 [.022 - .090]
**Sleep Disorder**	.300 [.232 - .368]	-.067 [-.102 - -.033]	.148 [.113 - .183]	.415 [.374 - .455]	.611 [.583 - .639]	.379 [.332 - .427]
**Income**	-.081 [-.093 - -.069]	-.257 [-.262 - -.252]	-.200 [-.206 - -.194]	-.062 [-.068 - -.055]	-.067 [-.071 - -.063]	.003 [-.005 - .011]
**Education**	-.129 [-.155 - -.103]	-.423 [-.433 - -.413]	-.237 [-.249 - -.226]	-.052 [-.067 - -.038]	-.040 [-.049 - -.031]	.100 [.081 - .108]

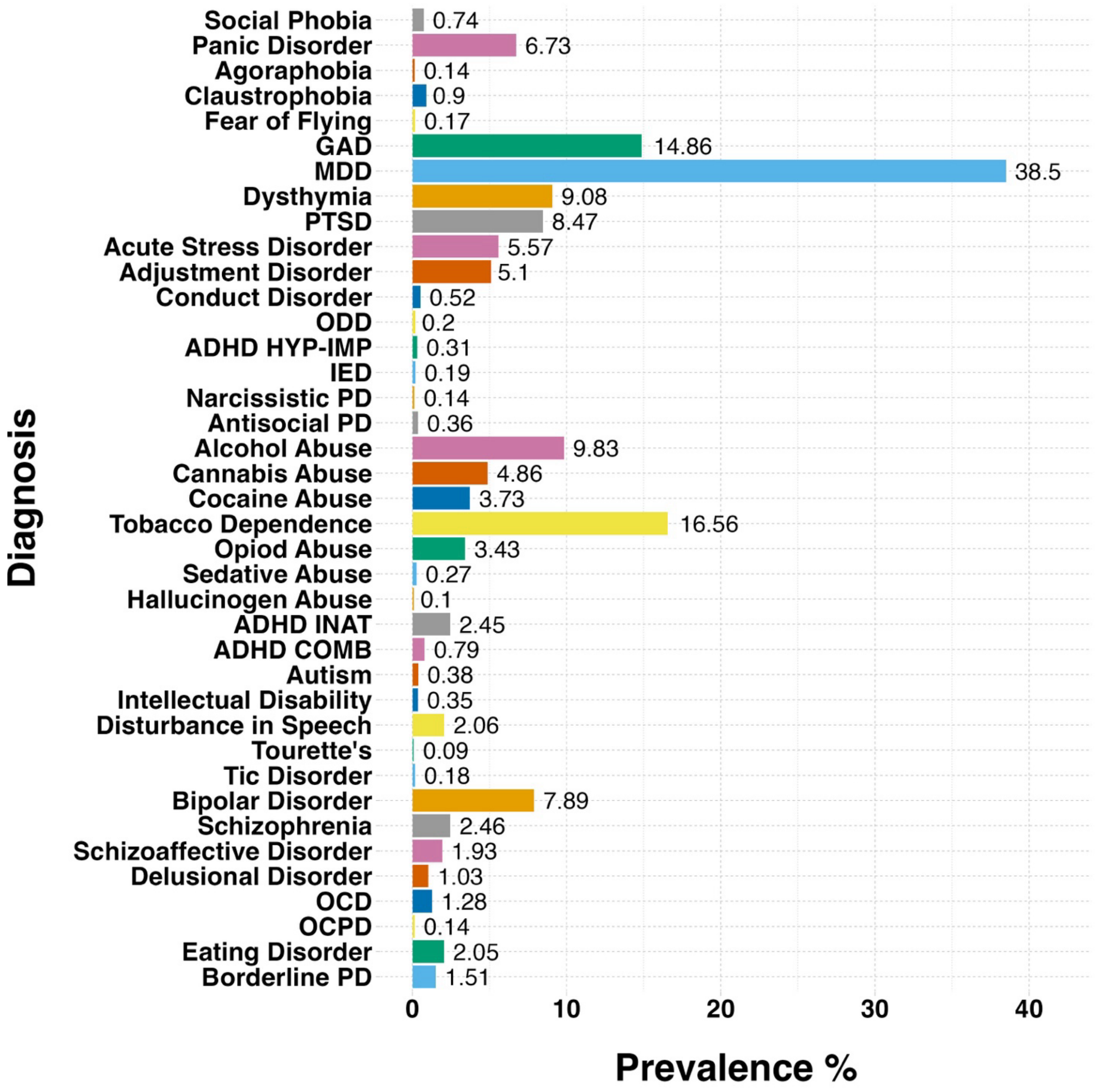

eFigure 1. *Prevalences of the 39 primary lifetime diagnoses*.

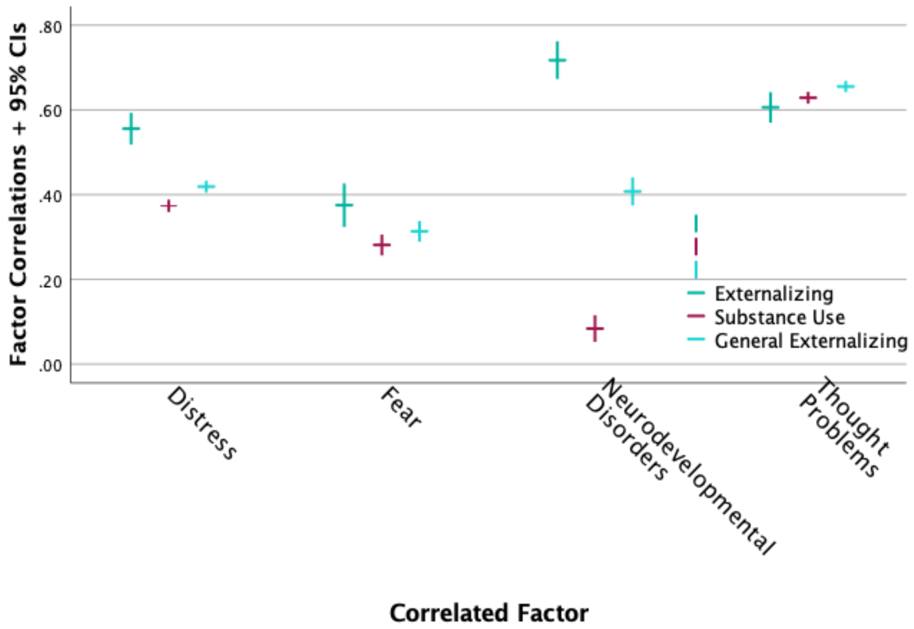

eFigure 2a. *Correlations of D, F, N, and T factors with Distinct Externalizing, Substance Use, and Combined Externalizing + Substance Use factors (General Externalizing)*

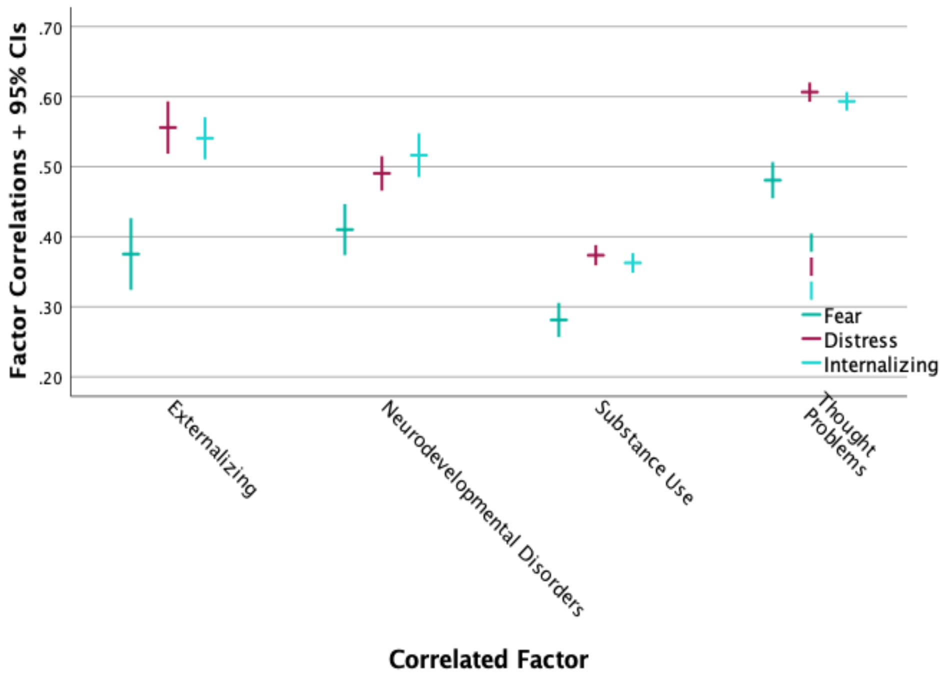

eFigure 2b. *Correlations of E, N, S, and T factors with Fear, Distress, and a broad Internalizing factor*

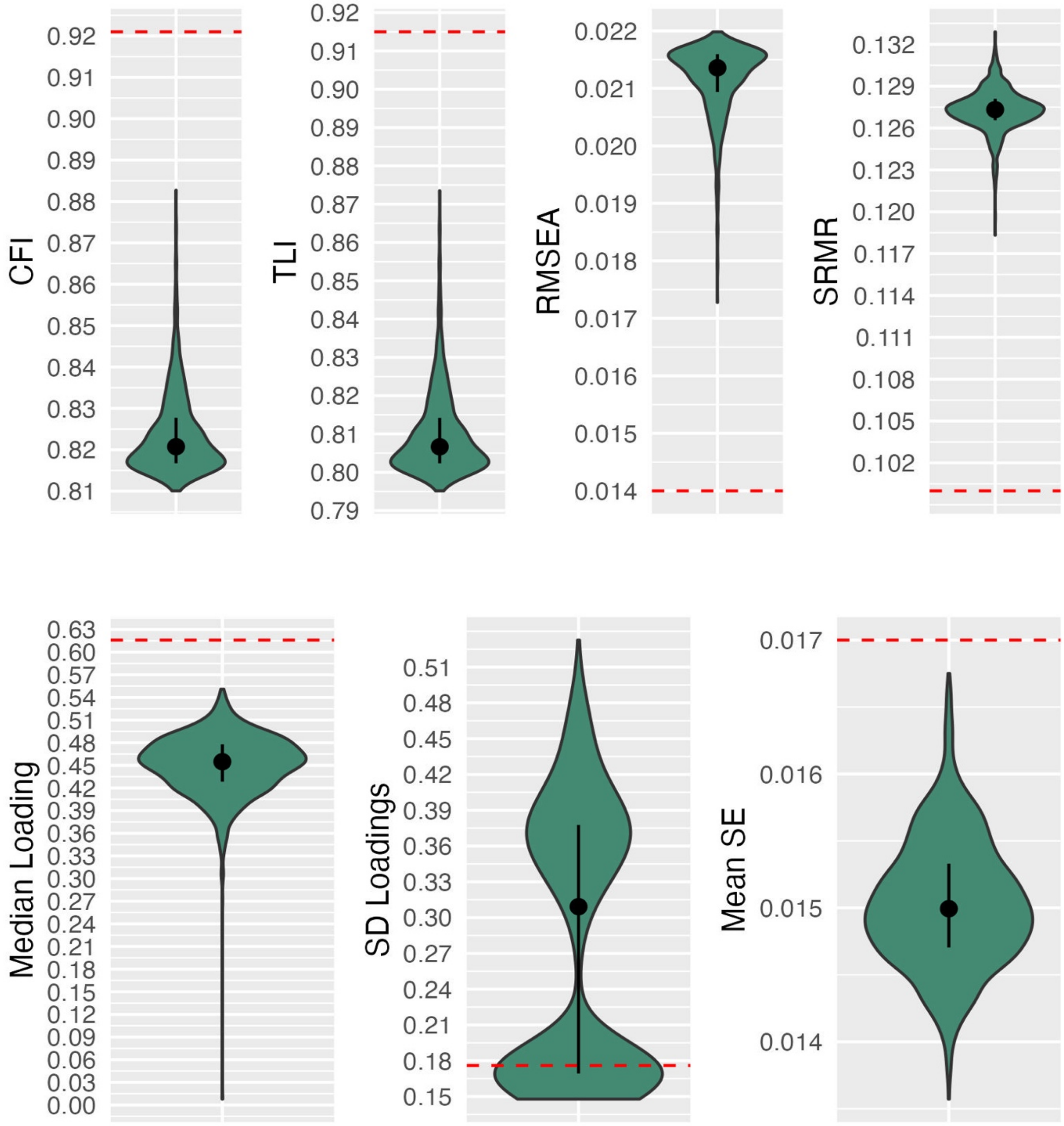

eFigure 3. *Indices of robustness of the results of the best-fitting model using random models***.**
Note. Red dashed line represents the fit of the best fitting CFA model (Model 9.5 with ASPD on S). The violins represent the distribution of the indices from the 1,000 random models.
